# Supplementary material for: Chromothripsis during telomere crisis is independent of NHEJ, and consistent with a replicative origin
Source: Genome Res. 2019 May;29(5):737–49. doi: 10.1101/gr.240705.118 (PMC6499312; doi:10.1101/gr.240705.118)
Supplement: Supplemental Material [file supp_gr.240705.118_Supplemental_file_1.zip › contigs/annotated_contigs/DB75/contig.2.DB75_length_641_mean_cov_7.95631825273.docx]

**DB75_length_641_mean_cov_7.95631825273**

```
TATACATATAAATAATATATATAATATACATAGAAACATGCAATATAACAAAATGGAATCACAAGGAAAGAGAATGTCTGAAAATACTT
 >chr10:110332922-110333279 + E=4e-203 p=1e-02
ATAGCTAGTAAAGAGATTCAATCAGTAGTCAAAAACCTTCCAACAAAGAAAAGCACAAGGCCTGTCTTCACTGAAGAATTCTATCAAAT

ATTCTTAAAAGAATGAATGCCAAGCCTTTTCAAATTCTTGCAATAATTGAATTAGATGGTATACTTCCAAATTCATTATATGAAGCCAG

TATTACCCTGATACCAAAGTCAGATAAAGATACTGCATGAAAAGAAGGCTACAGTTTAATATTTCTCATGAATATATGTCAAAATTGT|

TA|TATATATATATAAATGATTAATGATCCCATTTGCCTGAAATATATGTTATGTATATGTTATCCACATATAACATATACAGATGTTA
>chr10:110333843-110334129 + E=2e-157
TATATGTCATTTGTGTTTTATATGTTATATGTTATATATGTTTCTACAGATAAAACACACACACAAGGGAATATTATTCAGCCTTATAA

AAGAAGGAAATCTGTCACATGCTACATCATGAATGAATCTTGAGGATATCATGCTAAGCAAGATAAACCAGTGACAGAAAAATGAACAC

TGTAAGACTCCACTTTTATG
```
